# An improved multi-input deep convolutional neural network for automatic emotion recognition

**DOI:** 10.3389/fnins.2022.965871

**Published:** 2022-10-04

**Authors:** Peiji Chen, Bochao Zou, Abdelkader Nasreddine Belkacem, Xiangwen Lyu, Xixi Zhao, Weibo Yi, Zhaoyang Huang, Jun Liang, Chao Chen

**Affiliations:** ^1^Key Laboratory of Complex System Control Theory and Application, Tianjin University of Technology, Tianjin, China; ^2^School of Computer and Communication Engineering, University of Science and Technology, Beijing, China; ^3^Department of Computer and Network Engineering, College of Information Technology, United Arab Emirates University (UAEU), Al Ain, United Arab Emirates; ^4^National Engineering Laboratory for Risk Perception and Prevention, Beijing, China; ^5^Beijing Key Laboratory of Mental Disorders, Beijing Anding Hospital, Capital Medical University, Beijing, China; ^6^Beijing Machine and Equipment Institute, Beijing, China; ^7^Department of Neurology, Xuanwu Hospital, Capital Medical University, Beijing, China; ^8^Department of Rehabilitation, Tianjin Medical University General Hospital, Tianjin, China; ^9^Academy of Medical Engineering and Translational Medicine, Tianjin University, Tianjin, China

**Keywords:** biological signals, multi-modality, emotion recognition, convolutional neural network, machine learning

## Abstract

Current decoding algorithms based on a one-dimensional (1D) convolutional neural network (CNN) have shown effectiveness in the automatic recognition of emotional tasks using physiological signals. However, these recognition models usually take a single modal of physiological signal as input, and the inter-correlates between different modalities of physiological signals are completely ignored, which could be an important source of information for emotion recognition. Therefore, a complete end-to-end multi-input deep convolutional neural network (MI-DCNN) structure was designed in this study. The newly designed 1D-CNN structure can take full advantage of multi-modal physiological signals and automatically complete the process from feature extraction to emotion classification simultaneously. To evaluate the effectiveness of the proposed model, we designed an emotion elicitation experiment and collected a total of 52 participants' physiological signals including electrocardiography (ECG), electrodermal activity (EDA), and respiratory activity (RSP) while watching emotion elicitation videos. Subsequently, traditional machine learning methods were applied as baseline comparisons; for arousal, the baseline accuracy and f1-score of our dataset were 62.9 ± 0.9% and 0.628 ± 0.01, respectively; for valence, the baseline accuracy and f1-score of our dataset were 60.3 ± 0.8% and 0.600 ± 0.01, respectively. Differences between the MI-DCNN and single-input DCNN were also compared, and the proposed method was verified on two public datasets (DEAP and DREAMER) as well as our dataset. The computing results in our dataset showed a significant improvement in both tasks compared to traditional machine learning methods (*t*-test, arousal: *p* = 9.7E-03 < 0.01, valence: 6.5E-03 < 0.01), which demonstrated the strength of introducing a multi-input convolutional neural network for emotion recognition based on multi-modal physiological signals.

## Introduction

As an important component of artificial intelligence, affective computing plays an essential role in human–computer interaction (HCI). Emotion is the attitude and experience of people toward objective things; it integrates the state of human feelings, thoughts, and behaviors. When external stimuli cause changes in people's emotions, their physiological responses will change accordingly. The purpose of emotion recognition is to build a harmonious HCI environment by empowering the computer with human emotion capabilities and making the HCI process more intelligent. Research in the field of emotions can be traced back to the nineteenth century when American psychologist William James and Danish physiologist Carl Lange independently proposed the oldest theories of emotion (James, 1922), which supposed that emotional experience was the result of peripheral physiological changes caused by external stimuli. In the current stage, there are many emotion recognition tasks based on facial images (Akhand et al., 2021) and human speech signals (Chourasia et al., 2021; Mustaqeem and Kwon, 2021), which have achieved great development. Simultaneously, according to the maturity of wearable sensor technology, people have paid more and more attention to emotion recognition based on physiological signals, different from facial expressions and speech signals, and most changes in physiological signals cannot be voluntarily controlled, cannot be hidden, and can better reflect people's true emotional state. Furthermore, recording physiological signals can be continuous without interruption, which leads to feasible continuous emotional assessment. In this study, we collected subjects' multi-modal physiological signals, including ECG, EDA, and RSP through an emotion elicitation experiment. ECG reflects small electrical changes on the surface of the skin each time myocardial cells depolarize in the heartbeat, Agrafioti et al. (2012) found that when the induction method is active for subjects, ECG has a higher chance of responding to emotion, which proves the feasibility of using ECG for emotion recognition. EDA is a change in the electrical characteristics of the skin caused by the activity of sweat glands, which can reflect the change in arousal (Lang et al., 1993; Cacioppo et al., 2007). Respiration activity can reflect a person's stress states. When people are in a relaxed or pleasant state, their breathing rate will be relatively gentle, and when in a stressful or frightening state, their breathing rate could change rapidly or even stop. It has been proven in previous studies that the use of multi-modal physiological signals makes up for the limitation of the single modal in emotion recognition (Verma and Tiwary, 2014); applications based on multi-modal physiological signals are also increasing, such as emotion recognition (Zhang X. et al., 2021) and wearable health device (Cosoli et al., 2021). However, there are also disadvantages of physiological signal-based emotion recognition mainly because the difficulty of acquiring physiological signals is much higher than that of facial expressions and speech signals, which also limits the development of this field.

In recent years, the advancement of deep learning promoted the study of emotion recognition (Abbaschian et al., 2021; Makowski et al., 2021; He et al., 2022; Liu et al., 2022). In comparison with traditional machine learning, the use of deep learning for emotion recognition has a high improvement in accuracy and other aspects (Tang et al., 2017; Santamaria-Granados et al., 2019). Research shows that both convolutional neural networks and recurrent neural networks can achieve good results on emotion recognition tasks. Long short-term memory (LSTM) recurrent neural networks can extract context information from input sequences and avoid the long-term dependency problem. Zhang et al. (2017) used a deep learning framework to perform classification tasks on valence and arousal and confirmed its great potential in emotion recognition. Song et al. (2019) weakened the weight of certain useless sequences in the process of emotion classification by adding an attention mechanism to the LSTM network. Research on emotion recognition based on convolutional neural networks (CNNs) using physiological signals has the following two paths: the first is to transform physiological signals into spectrograms and then two-dimensional (2D) convolution is used for feature extraction. 2D convolutional neural networks have a very mature structure, which is beneficial for extracting physiological signal features from 2D images. For instance, Siddharth et al. (2022) used a pre-trained VGG-16 network to extract features from the spectrogram, and then they used a long short-term memory (LSTM) network for classification. The second one is to use a one-dimensional (1D) convolutional neural network to directly classify raw physiological signals. For example, in the research of Zhang Y. et al. (2021), they used a 1D convolutional neural network to extract efficient features from biological signals; this structure can automatically complete the feature extraction and classification of physiological signals. Unlike using 2D convolutional neural networks, the 1D-CNN does not require many preprocesses and can be deployed in an end-to-end manner. However, previous research on emotion recognition based on 1D convolutional neural networks usually utilizes a single modal physiological signal, and even if it is based on multi-modal, it is only a decision-level fusion, not a feature-level fusion. Therefore, we proposed an MI-DCNN, which enables the feature-level fusion of multi-modal physiological signals for emotion recognition.

This study focuses on using multi-modal physiological signals for automatic emotion recognition, and the difference between single-input and multi-input in the CNN will also be discussed. Simultaneously, we evaluate our proposed method on two public datasets and our collected data. This article consists of five sections. Section Related research provides an overview of related research. Section Introduction of our emotional dataset introduces the experimental process and the preprocess methods used in our dataset. Our proposed method is presented in Section Methods. We also give a baseline result of our dataset and evaluation of our proposed method in Section Evaluation, and we then draw conclusions in the last section.

## Related research

The development of emotion recognition research based on multi-modal physiological signals mainly benefits from the establishment of physiological emotion datasets and the rise of deep learning techniques. The research of Gross and Levenson (1997) shows that audiovisual stimuli can better induce participants' emotions; we, therefore, summarized some popular physiological emotion datasets and related research in Table 1, where all datasets use audiovisual stimuli as elicitation materials.

**Table 1:** Summary of the multi-modal physiological research using video stimulation.

**Dataset**	**References**	**Modalities**	**Feature extraction**	**Emotion label**	**Classifier**	**Evaluation**
ASCERTAIN	Subramanian et al., 2018	ECG, GSR, EEG, EMO	Multiple physiological features and facial action units	Valance/arousal	Non-linear statistics and RBF SVM	64%/62% for valance and arousal with peripheral Signals (ECG + GSR) (2-classes)
DECAF	Abadi et al., 2015	EEG, MEG, NIR, hEOG, ECG, tEMG	Multiple physiological features and audio-video features	Valance/arousal	Linear SVM	56%/60% for valance and arousal with peripheral physiological (2-classes)
DREAMER	Katsigiannis and Ramzan, 2018	EEG, ECG	HRV, PSD	Valance/arousal	SVM	61.84%/62.32% for Valance/Arousal using all modalities (2-classes)
	Siddharth et al., 2022	EEG, ECG	PSD, HRV	Valance/arousal	LSTM	79.95% Valance/Arousal using all fusion(2-classes)
DEAP	Wang and Shang, 2013	EEG, EOG, EMG	Raw data	Valance/arousal	DBN	51.2%//60.09% Arousal/Valence (2-classes)
	Tripathi et al., 2017	EEG	Image features extracted by convolutional neural networks	Valance/arousal	DNN/CNN	81.41%/73.36% for arousal and valence using EEG (2-classes)
	Siddharth et al., 2022	EEG, ECG, GSR	Physiological features and features extracted by pre-trained VGG-16 model	Valance/arousal	LSTM	71.87%/73.05% for valance and arousal (2-classes)
AMIGOS	Santamaria-Granados et al., 2019	ECG, GSR	Time-domain-non-linear features, mean, min, max, standard deviation etc.	Valance/arousal	DCNN	75%/76% for valance and arousal using all modalities (2-classes)
	Miranda-Correa et al., 2021	EEG, ECG, GSR	Multiple physiological features	Valance/arousal	Gaussian Bayes/SVM	56%/56.4% for valance and arousal using all modalities (2-classes)
MAHNOB-HCI	Siddharth et al., 2022	EEG, ECG, GSR	PSD, HRV, Statistical features in time- frequency domain.	Valance/arousal	LSTM	80.36%/80.61% for valance and arousal using peripheral physiological features (2-classes)
	Subramanian et al., 2018	ECG	Statistical distributions of dominant frequencies (DFs) from IMFs and their difference	Valance/arousal	KNN	59.2%/58.7% for valence and arousal (3-classes)
MPED	Song et al., 2019	EEG, GSR, RSP, ECG	PSD, STFT, HHS, HOC, Hjorth (1970)	Joy, funny, anger, fear, disgust and neutrality	KNN/SVM/LSTM/A-LSTM	Several protocols (Song et al., 2019)

The modalities include electrocardiography (ECG), electroencephalogram (EEG), galvanic skin response (GSR), horizontal electrooculography (hEOG), electromyography (tEMG), and respiratory activity (RSP). The feature extraction methods include heart rate variability (HRV), power spectral density (PSD), short-time Fourier transform (STFT), Hilbert–Huang spectrum (HHS), higher order crossing (HOC), and intrinsic mode functions (IMFs). VGG-16: Visual Geometry Group Network (consists of 13 convolutional layers and three fully connected layers). The classifiers include support vector machine (SVM), K-nearest neighbors (KNN), deep neural network (DNN), deep convolutional neural network (DCNN), long short-term memory (LSTM), attention-long short-term memory (A-LSTM), and deep belief network (DBN).

Previous research studies (Kolodyazhniy et al., 2011; He et al., 2020) have demonstrated the potential of fusion of multi-modality for emotion recognition tasks. Subramanian et al. (2018) recorded electroencephalogram (EEG), electrocardiogram (ECG), and galvanic skin response (GSR) of 58 subjects during watching the video and used physiological features for emotion and personality trait recognition. Abadi et al. (2015) presented a dataset to estimate subjects' physiological responses to elicitation materials using brain signals and several physiological signals from 30 participants. Katsigiannis and Ramzan (2018) recorded EEG and ECG signals of 23 subjects using wearable, low-cost devices and showed the broad prospects of using these devices for emotion recognition. Soleymani et al. published MAHNOB-HCI (Soleymani et al., 2012) and DEAP (Koelstra et al., 2012) to explore the relationship between human physiological responses and emotional states. Miranda-Correa et al. (2021) recorded 40 subjects' EEG, ECG, and GSR signals elicited by visual stimuli for multi-modal research of emotion states on individuals and groups. MPED (Song et al., 2019) consists of EEG, GSR, RSP, and ECG signals of 23 participants while watching videos, and an attention-long short-term memory (A-LSTM) network was designed for emotion recognition. Siddharth et al. (2022) used deep learning methods for physiological signal-based emotion recognition on four public datasets (Koelstra et al., 2012; Soleymani et al., 2012; Katsigiannis and Ramzan, 2018; Miranda-Correa et al., 2021) and demonstrated the superiority of deep learning methods.

As a structure of a deep learning network, the convolutional neural network is mostly used in image classification and other related fields and has made great achievements. However, the 1D convolutional neural network, with its special structure, is widely used to process time series signals. Previous studies (Acharya et al., 2018; Yildirim et al., 2018) had proved the superiority of using the 1D convolutional neural network to classify time series signals. Yildirim et al. (2018) designed a 16-layer CNN model to detect 17 types of arrhythmia diseases and reached an overall classification accuracy of 91.33%. Acharya et al. (2018) used a 13-layer DCNN with an EEG signal to classify normal, preictal, and seizures, which achieved an accuracy of 88.76%. In the field of physiological signal-based automatic emotion recognition, the 1D convolutional neural network also achieved better results. Santamaria-Granados et al. (2019) used 1D deep convolutional neural networks for emotion recognition on the AMIGOS dataset and achieved a great improvement compared with previous research. Several models based on 1D convolutional neural networks are used for automatic emotion recognition, and end-to-end characteristics and better recognition results also prove the advantages of using such models. However, current research on 1D-CNN-based emotion recognition usually used a single-modal physiological signal as input, which neglects the inter-correlates between different modalities of physiological signals; therefore, in this article, we proposed an MI-DCNN for linking the relationship between multi-modalities.

## Introduction of our emotional dataset

### Emotion elicitation experiment

A total of 52 subjects were recruited to participate in our experiment. We elaborately chose 12 videos as elicitation materials, which are shown in Table 2. All of these video materials run 34–201 s and contain different emotional states, such as fear, disgust, sadness, anger, and happiness. Before the formal experiment, the participants need to complete the Patient Health Questionnaire-9 (PHQ-9; Kroenke and Spitzer, 2002) to evaluate their recent emotional state. The participants who do not reach the specified score are not allowed to participate in the experiment, and those who reach the specified score were given MP150 physiological signal recorders in the shielded room to randomly watch the videos, and then three physiological signals (ECG, EDA, and RSP) were collected. There is a piece of 30-s music between every two videos to calm their moods, and the emotion evaluation scheme was the Self-Assessment Manikin (SAM; Morris, 1995). After watching each video, the participants filled in the evaluation according to their current feelings, and the valuation options mainly include the arousal level (discrete value of 1-9), valence level (discrete value of 1-9), and familiar level (-1, 0, 1). Because the nine-point scale was used, a threshold was placed in the middle, which leads to unbalanced classes, also reported by the studies of the first two public datasets (Koelstra et al., 2012; Katsigiannis and Ramzan, 2018).

**Table 2:** Details of the emotion elicitation material in our dataset.

**Emotion**	**Video name**	**Description**	**Duration**	**Video name**	**Description**	**Duration**
Disgust	FOOD FOR LOUIS	A man eats mealworms	34 s	FOOD FOR LOUIS	A man eats cockroaches	42 s
Fear	Myopia surgery	Doctor does eye surgery for a patient	116 s	Ring	A woman crawled out of the television	201 s
	Final destination	A serious car accident	103 s	The eye	A woman meets a ghost in the elevator	77 s
Sadness	Redmond's Olympic Story	An athlete finishes the race with injury	91 s	Wenzhou train collision	Families of the accident seek the truth	117 s
Anger	Beating gravida	A man beats a woman in a restaurant	144 s	Dog abuse	A man abetted a big dog to bite a little dog	96 s
Happiness	Larva farting episode	Yellow tries to prevent the fart from coming out	92 s	Fault funny collection	fragments of mistakes and funny accidents	77 s

In addition, each subject will first collect baseline data in the initial state of 60 s, which are used to compensate for data differences caused by individual differences (Wagner et al., 2005). After the trial, the participants watched their recorded video and marked the stimulus segments. Finally, two psychologists evaluated the videos of all participants and cut out the needed parts.

### Feature extraction from physiological signals

**Figure 1:**
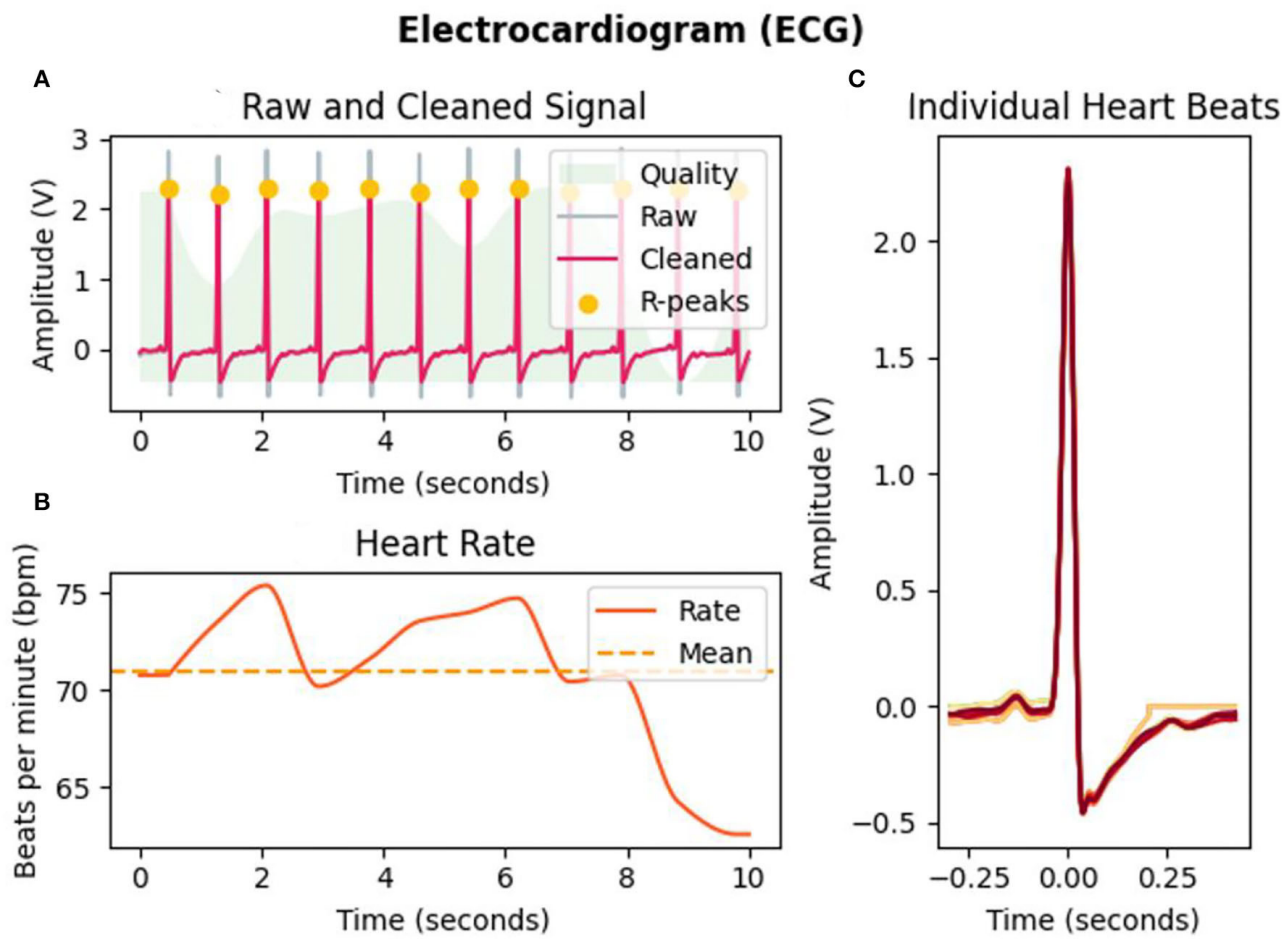
ECG analysis of subject 1 in the selected interval (10 s) of video b. **(A)** Filtering and R-peaks detection of ECG signals. **(B)** Heart rate changes during the selected interval. **(C)** Individual heartbeats.

The physiological signal has weak amplitudes and high susceptibility to noise and magneto- and electro-interference. Different source signals come from different parts of the body. Therefore, different preprocesses of raw signals are needed. We use the Neurokit2 Toolbox (Makowski et al., 2021) to process the raw biological signals. ECG signals are mostly concentrated between 0 and 50 HZ, and a finite impulse response (FIR) band-pass filter between 3 and 45 Hz is used to filter the raw ECG signal, as shown in Figure 1A where the gray line represents the raw ECG signal and the pink line represents the filtered ECG signal. Then, filtered data are passed through Hamiltonian segmentation (Hamilton, 2002) to detect the QRS complex of each heartbeat, the heartbeat changes while watching the elicitation materials are recorded as Figure 1B shown, and individual heartbeats during this period are shown in Figure 1C. Finally, the R-peaks are identified as the orange points, as shown in Figure 1A. Extraction of electrodermal activity (EDA) is based on convex optimization (Greco et al., 2016). For respiration, statistical characteristics were calculated, such as mean, maximum, and variance value of the intensity of each breathing cycle. From the three physiological signals collected in our emotion elicitation experiment, 58 features were extracted for traditional machine learning methods, mainly including 47 ECG features (Table 3), four EDA features, and seven RSP features.

**Table 3:** ECG features used in this research.

	**Feature name**
Time domain	RMSSD, MeanNN, SDNN, SDSD, CVNN, CVSD, MedianNN, MadNN, MCVNN, IQRNN, pNN50, pNN20, TINN, HTI
Frequency domain	HF, VHF, HFn, LnHF
Non-linear domain	SD1, SD2, SD1SD2, S, CSI, CVI, CSI Modified, PIP, IALS, PSS, PAS, GI, SI, AI, PI, C1d, C1a, SD1d, SD1a, C2d, C2a, SD2d, SD2a, Cd, Ca, SDNNd, SDNNa, ApEn, SampEn

## Methods

A multi-input deep convolutional neural network for emotion recognition based on multi-modal physiological signals is designed and compared with the previous DCNN model. Our proposed multi-input deep convolutional neural network can extract the features of different input signals separately, and simultaneously, the filters in different channels are not shared, which guarantees that each input can have a different convolutional setting for the different input signals. The multi-input deep convolutional neural network proposed in this research can be regarded as a data fusion method at the feature level, and each branch in this structure can rely on the 1D-CNN to extract features automatically from the input signals without the need for manual feature extraction and selection, and the feature extracted from each input will be concatenated together through the concatenate layer. Then, the feature vector will be sent to the fully connected network for feature fusion and selection. This efficient feature-level fusion method does not base on any prior knowledge or complex feature engineering, and it can automatically fuse the most distinguishing features from each channel and eliminate redundant features between each channel. The whole process is end-to-end, which is more conducive to real-time subsequent decision-making. In our designed multi-input deep convolutional neural network, each channel inputs one modal of physiological signal; therefore, by adding input channels, the model can take multi-modal physiological signals, instead of a single modal, which may have potential benefit for emotion recognition.

**Figure 2:**
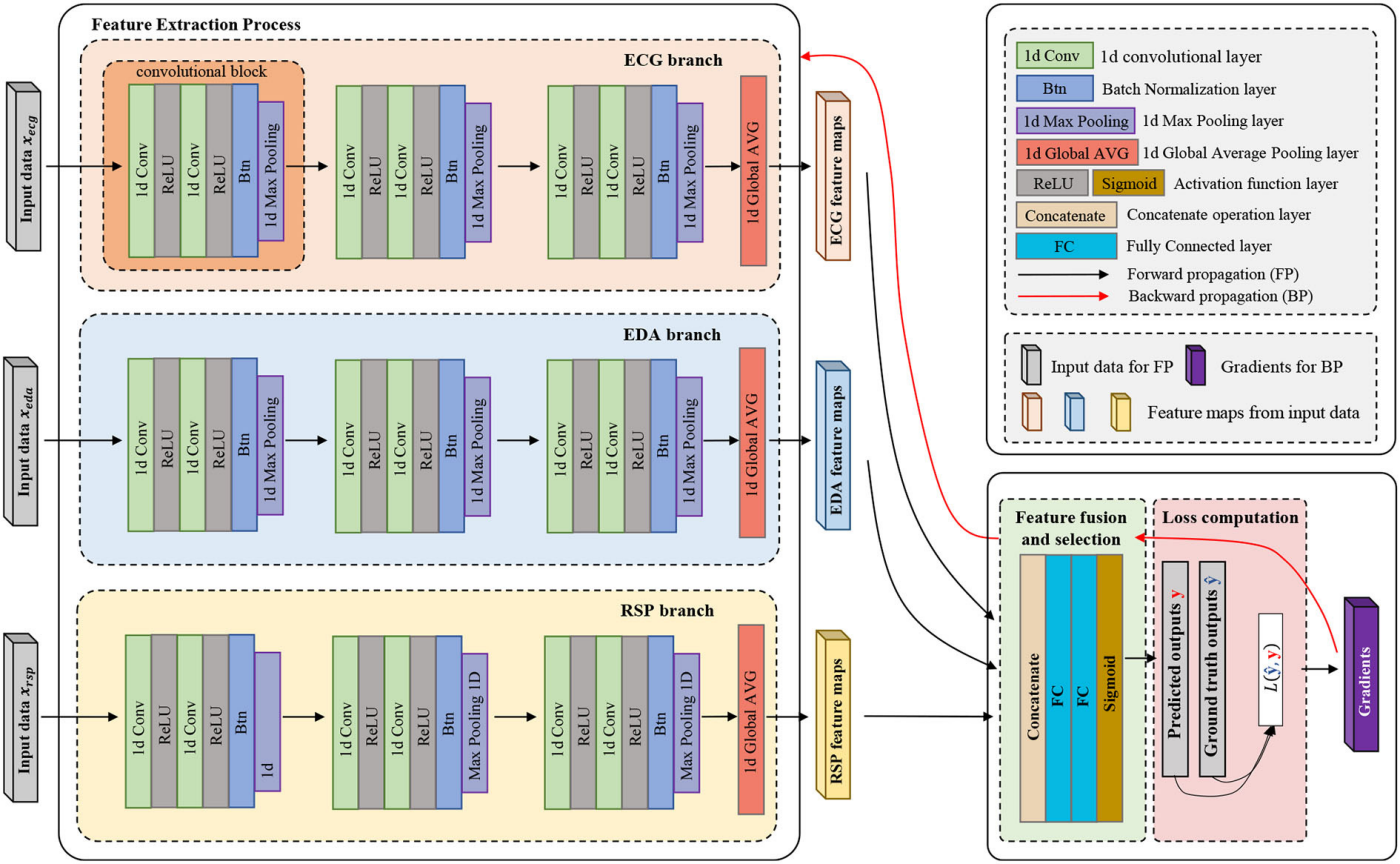
Multi-input deep convolutional neural network.

The structure of the model is mainly composed of convolution layers, pooling layers, and fully connected layers. Figure 2 illustrates the overall structure of the multi-input deep convolutional neural network designed in this study, and Table 4 details the parameter setting of the automatic feature extraction structure used in our model.

**Table 4:** Detailed feature extraction structure of the multi-input deep convolutional neural network.

**Layer name**	**Filters** × **kernel size**			**Other layer parameters**
	**Input-1**	**Input-2**	**Input-3**	
Conv Block1	$\left[\begin{array}{c} 128\times 32\\ 128\times 32\\ -\\ - \end{array}\right]$	$\left[\begin{array}{c} 64\times 16\\ \;64\times 16\\ -\\ - \end{array}\right]$	$\left[\begin{array}{c} 128\times 16\\ 128\times 16\\ -\\ - \end{array}\right]$	Activation = ReLU, Strides = 2/2/3, Padding =”'same” Activation = ReLU, Strides = 2/2/3, Padding = “same” - Pooling size = 2
Conv Block2	$\left[\begin{array}{c} 128\times 32\\ \;128\times 32\\ -\\ - \end{array}\right]$	$\left[\begin{array}{c} \;64\times 16\\ \;64\times 16\\ \;-\\ - \end{array}\right]$	$\left[\begin{array}{c} \;64\times 16\\ \;64\times 16\\ \;-\\ - \end{array}\right]$	Activation = ReLU, Strides = 2/3/2, Padding = “same” Activation = ReLU, Strides = 1/3/2, Padding = “same” - Pooling size = 2
Conv Block3	$\left[\begin{array}{c} \;128\times 16\\ \;128\times 16\\ \;-\\ - \end{array}\right]$	$\left[\begin{array}{c} \;32\;\times \;4\\ \;32\;\times \;4\\ \;-\\ - \end{array}\right]$	$\left[\begin{array}{c} \;64\;\times \;8\\ \;64\;\times \;8\\ \;-\\ - \end{array}\right]$	Activation = ReLU, Strides = 1/1/1, Padding = “same” Activation = ReLU, Strides = 1/1/1, Padding = “same” - Pooling size = 2
GAP layer	[(?, 128)]	[(?, 32)]	[(?, 64)]	-
Concatenate layer	[(?, 224)]			-

GAP, global average pooling. ? means batch size.

**Figure 3:**
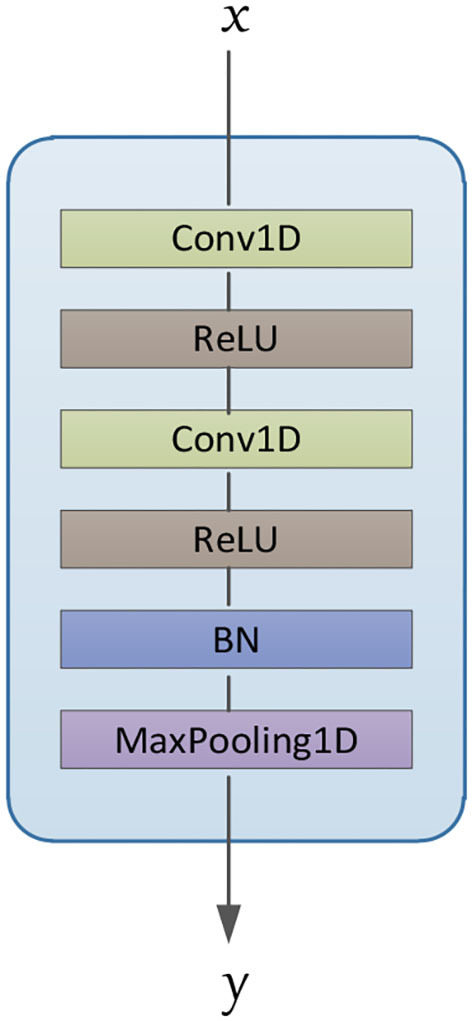
Architecture of convolutional blocks.


The model is divided into three parts according to its functionality (convolutional, concatenate, and fully connected layers). Convolutional layers play the role of feature extraction, concatenate layer will concatenate the features of each channel from deep convolution layers, and fully connected layers get emotion class by these features. As shown in Figure 2, in each processing channel, multiple convolution blocks are stacked to form a deep convolution structure. The composition structure of the convolutional blocks is shown in Figure 3, which consists of four parts: (1) 1D convolutional layer, (2) activation function, (3) batch normalization layer, and (4) max-pooling layer.

(1) One-dimensional convolutional layer: It consists of fixed-number and fixed-size filters (convolution kernels). Each filter can be regarded as a fuzzy filter (Zhang Y. et al., 2021), and it slides on the input signal or is a result of the previous layer, and performs the convolution operation according to Equation 1:

(1) $$x_{j}^{k}=\sum_{i\in M_{j}}w_{ij}^{k*}x_{i}^{k-1}+b_{j}^{k}$$

Where $x_{i}^{k-1}$ represents the input vector of this convolutional layer, *M*_*j*_ indicates the size of the receptive field, and $w_{ij}^{k}$, $b_{j}^{k}$ are two trainable parameters, where $w_{ij}^{k}$ denotes the kernel weight between the *i*^*th*^ neuron in the *k*−1 layer and the *j*^*th*^ neuron in the *k* layer; $b_{j}^{k}$ denotes the bias coefficient of the *j*^*th*^ neuron in the *k* layer; and $x_{j}^{k}$ represents the output vector corresponding to the *j*^*th*^ convolution kernel in the *k* layer.

(2) Activation function: We add an activation function after each convolutional layer, and the activation function can increase the non-linearity of the model and improve the expression ability of the model. There are many types of activation functions. In order to prevent the vanishing gradient problem, we choose the rectified linear unit (ReLU) as the activation function. Equation 2 shows the ReLU function.

(2) $$f\left(x\right)=\{\begin{array}{cc} x,\; & x>0\\ 0,\; & otherwise \end{array}$$

(3) Batch normalization: This method has been proposed by Ioffe and Szegedy (2015), which is based on adjusting the activation value distribution of each layer to have the appropriate breadth, eliminating the problem of internal covariate shift. Simultaneously, the addition of the batch normalization layer can also accelerate the learning process and suppress overfitting. This can be expressed in a mathematical formula as follows:

(3) $$\begin{array}{l} \mu_{B}\leftarrow \frac{1}{m}\overset{m}{\underset{i=1}{\sum^{\text{​}}}}x_{i} \end{array}$$

(4) $$\begin{array}{l} \sigma_{B}^{2}\leftarrow \frac{1}{m}\overset{i=1}{\underset{m}{\sum }}{\left(x_{i}-\mu_{B}\right)}^{2} \end{array}$$

(5) $$\begin{array}{l} {\hat{x}}_{i}\leftarrow \frac{x_{i}-\mu_{B}}{\sqrt{\sigma_{B}^{2}+\varepsilon }} \end{array}$$

(6) $$\begin{array}{l} y_{i}\leftarrow \gamma {\hat{x}}_{i}+\beta \end{array}$$

where μ_*B*_ and ${\;\sigma }_{B}^{2}$ represent the mean and variance of the data input to the batch normalization layer. Equation 5 indicates that the input data are regularized with a mean value of 0 and a variance of 1. In order to prevent the situation of dividing by 0, a minimum value ε is added. Equation 6 represents the scaling and translation transformation of regularized data; ${\hat{x}}_{i}$, γ, and β are parameters, which will be adjusted to appropriate values by the training of the model.

(4) Max-pooling layer: A max-pooling layer is added at the end of each convolution block. The max-pooling layer removes redundant information from the input vector and extracts important features by taking the maximum value of the input vector within the set range. Simultaneously, the complexity and calculation of the model are reduced, and overfitting is prevented.

A global average pooling layer is added at the end of the deep convolution structure of each channel. It is an important step to achieve multiple inputs. Through this step, the features extracted from different channels can be concatenated. We also evaluated that the global average pooling layer was added behind the concatenate layer, but in this case, it is necessary to ensure that the shape of the feature map output by each channel must be consistent, which also limits the structure of the model. Finally, the prediction results are output through two fully connected layers. During the model training process, the Adam (Kingma and Ba, 2015) optimizer is selected for parameter update, and cross-entropy is used as the loss function. The used framework is TensorFlow (Abadi et al., 2016). The network is trained on a GTX2080Ti GPU. The dataset is divided into two parts (train dataset and test dataset), with a division ratio of 80/20, and a 5-fold cross-validation was performed.

## Evaluation

In this article, we will use three traditional machine learning methods (support vector machines, random forests, K-nearest neighbors) and deep learning methods to analyze our dataset and public datasets, including the classification of valence and arousal.

**Figure 4:**
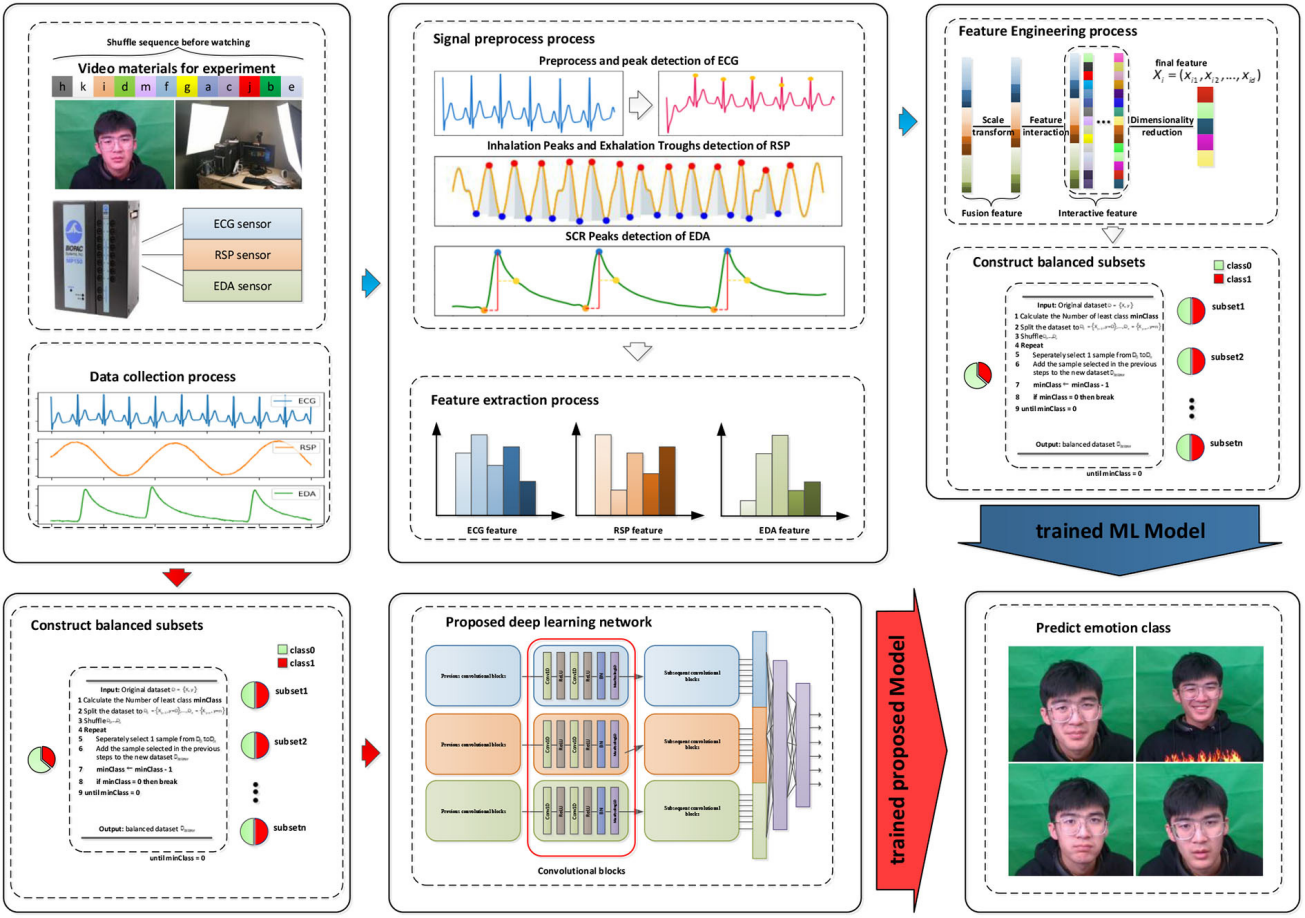
Framework of emotion recognition in our research.

The results obtained by the three traditional machine learning methods are used as the baseline data of our dataset, which can be used for follow-up research. The machine learning method used in this study is from scikit-learn (Pedregosa et al., 2018). Simultaneously, a single-input DCNN is used to evaluate the three modalities together and separately, and the evaluation results, along with the results from our MI-DCNN, were used to investigate when the input signals are significantly different, and whether the multi-input DCNN model performs better than the single-input DCNN model. Figure 4 shows the emotion recognition framework based on traditional machine learning methods and our proposed deep learning method, where the blue arrow represents the emotion recognition process based on traditional machine learning and the red arrow represents the emotion recognition process based on deep learning. For the traditional machine learning process, the following steps are required: (a) data collection from the subjects, (b) signal preprocessing and feature extraction for raw signals, (c) feature engineering and construction of balanced subsets, and (d) training the ML models and predicting the emotion class. For our proposed deep learning methods, the following steps are necessary: (a) data collection from the subjects, (b) construction of balanced subsets, (c) training the DL models, and predicting the emotion class. Compared with traditional machine learning methods, our proposed deep learning emotion recognition method eliminates the process of signal processing, feature extraction, and feature engineering, which is a complete end-to-end model.

## Baseline of our dataset

Considering our dataset is not a balanced dataset, we randomly created five fully balanced datasets on the current dataset according to the least type of categories to make our results more convincing. For each sub-dataset, 80% of the samples are used as the training set, the remaining 20% of the samples are used as the test set, and five-fold cross-validation was performed. The average of the results of the five sub-datasets is regarded as the result of the sentiment dataset.

We used single-modal physiological signals (ECG, EDA, and RSP) and multi-modal physiological signals for emotion recognition, respectively. While using traditional machine learning classifiers, we also used random classifiers and majority classifiers as baseline comparisons. Random classifier makes random predictions on samples, and the majority classifier is a model that makes predictions based on most samples. For the training process of the three traditional machine learning algorithms, the grid search was used to search and compare the inherent parameters of each machine learning model to select the best combination of parameters. The specific results of using traditional machine learning methods are shown in Table 5.

**Table 5:** Baseline of our dataset.

**Classifiers**	**Modality**	**Arousal**		**Valence**	
		**Accuracy**	**F1-score**	**Accuracy**	**F1-score**
SVM	ECG	60.0 ± 1.7%	0.599 ± 0.02	58.0 ± 1.1%	0.573 ± 0.02
	EDA	58.5 ± 1.3%	0.584 ± 0.01	55.3 ± 1.7%	0.512 ± 0.02
	RSP	59.2 ± 2.3%	0.579 ± 0.04	55.5 ± 1.4%	0.548 ± 0.01
	Fusion	62.9 ± 0.9%	0.628 ± 0.01	60.3 ± 0.8%	0.600 ± 0.01
RFC	ECG	61.4 ± 2.4%	0.612 ± 0.02	56.8 ± 1.1%	0.567 ± 0.01
	EDA	56.3 ± 1.2%	0.561 ± 0.01	54.6 ± 1.5%	0.535 ± 0.02
	RSP	59.9 ± 1.4%	0.595 ± 0.01	57.6 ± 1.1%	0.569 ± 0.01
	Fusion	62.9 ± 2.1%	0.627 ± 0.02	59.7 ± 1.3%	0.594 ± 0.02
KNN	ECG	59.5 ± 3.1%	0.592 ± 0.03	55.9 ± 2.1%	0.554 ± 0.02
	EDA	54.3 ± 2.1%	0.523 ± 0.02	53.6 ± 1.6%	0.520 ± 0.02
	RSP	58.9 ± 1.9%	0.588 ± 0.02	55.7 ± 2.1%	0.552 ± 0.02
	Fusion	62.7 ± 1.2%	0.625 ± 0.01	59.2 ± 0.7%	0.584 ± 0.01
Random		50.1 ± 0.2%	0.489 ± 0.01	49.9 ± 0.1%	0.489 ± 0.01
Majority		50.0%	0.333	50.0%	0.333

We analyzed the baseline results of the dataset and discussed the performance differences of emotion recognition based on single-modality and multi-modality physiological signals on different traditional machine learning classifiers. For arousal, the results of using the multi-modal had a significant improvement than single modal (*t*-test, ECG: 6.5E-04 < 0.01, EDA: 7.5E-04 < 0.01, RSP: 3.6E-05 < 0.01), and for valence, the results of using the multi-modal also showed a significant improvement than the single modal (*t*-test, ECG: 5.2E-06 < 0.01, EDA: 1.2E-04 < 0.01, RSP: 8.9E-04 < 0.01). The support vector machine algorithm achieved the best classification results in arousal classification tasks, with an accuracy of 62.9 ± 0.9% and an f1-score of 62.8 ± 1.0%. Compared with the results of arousal, the results of valence were relatively inferior at the level of the classifier or at the level of different modalities. The support vector machine algorithm also achieved the best results on the valence task, with an accuracy of 60.3 ± 0.8% and an f1-score of 60.0 ± 1.0%.

### Comparisons with the other public datasets

To evaluate the quality of our dataset, we compared the baseline results of our dataset with those of three public datasets. The comparison results are shown in Table 6. First, a brief introduction to the three public datasets was performed.

**Table 6:** Comparison with other public datasets.

**Datasets**	**Modality**	**Arousal**		**Valence**	
		**Accuracy**	**F1-score**	**Accuracy**	**F1-score**
DEAP	EEG	62.0%	0.583	57.6%	0.563
	Peripheral	57.0%	0.533	62.7%	0.608
	Majority class	64.4%	0.389	58.6%	0.368
DREAMER	EEG	62.2%	0.577	62.5%	0.518
	ECG	62.4%	0.580	62.4%	0.531
	Fusion	62.3%	0.575	61.8%	0.521
AMIGOS	EEG	N/A	0.577	N/A	0.564
	GSR	N/A	0.541	N/A	0.528
	ECG	N/A	0.551	N/A	0.545
	Fusion	N/A	0.564	N/A	0.560
OURS	ECG	61.4%	0.612	58.0%	0.573
	EDA	58.5%	0.584	55.3%	0.512
	RSP	59.9%	0.595	57.6%	0.569
	Fusion	62.9%	0.628	60.3%	0.600
	Majority class	50.0%	0.333	50.0%	0.333

#### DEAP

A total of 32 participants were recorded with EEG data, peripheral physiological data, frontal facial videos, and participants' self-assessment reports while watching emotion elicitation video stimuli. Considering the relevance of the study, only peripheral physiological signals and participants' self-assessment reports were analyzed in this study. In total, 40 emotional videos were selected as stimuli, and through an effective highlighting algorithm, the 60-s segment with maximum emotional content is detected. The emotional evaluation model uses the Self-Assessment Manikin (SAM; Morris, 1995), including valence, arousal, and dominance, in which the dominance scale ranges from submissive to dominant. The values of all three options are continuous values from 1 to 9; in addition, the division of valence/arousal degree is also strictly in accordance with the study mentioned in Koelstra et al. (2012).

#### DREAMER

Instead of medical-grade devices, the DREAMER dataset is used with low-cost, wearable devices. The stimuli materials were obtained from a dataset consisting of 18 videos evaluated by Gabert-Quillen et al. (2015). EEG, ECG, and self-assessment reports of 23 participants were recorded through the emotional eliciting experiment, and the length of stimuli videos ranges from 65 to 393 s. Only the data corresponding to the last 60 s of each video are clipped for research. Similar to DEAP, the emotional evaluation model also uses the Self-Assessment Manikin, but the scope scale is different.

#### AMIGOS

It is a multi-modal physiological signal dataset for personality characteristics and emotion recognition research of individuals and groups, using long and short video-inducted materials, collecting EEG signals, ECG signals, GSR signals, and facial videos of 40 participants. The emotional evaluation model is also a Self-Assessment Manikin.

Different from the other three datasets, we did not collect EEG data in our experiment, so we will not discuss EEG signals in detail. For peripheral physiological signals, the baseline results of arousal and valence in the DEAP dataset are lower than those in our dataset, which had an accuracy of 57 and 62.7%, and an f1-score of 0.533 and 0.608, respectively. In addition, the results in the DEAP dataset were not obtained by using balanced data, and from the majority class classifier, it can be seen that when the model predicts the test samples according to the proportion, the accuracy is even higher than that of the trained machine learning model, but the f1-score is very low. This is because the majority class classifier predicts the test set into the same class (the class with more samples in the training set). The DREAMER dataset uses EEG and ECG to perform the emotion recognition task, which had accuracies of arousal and valence of 62.4 and 62.4%, and f1-scores of arousal and valence of 0.580 and 0.531. The AMIGOS dataset only calculated the results of the f1-score, which were slightly lower than our results when using fusion peripheral data. In our dataset, we first construct a fully balanced dataset and then judge on the balanced dataset so that the results obtained will not be falsely high, from the majority class classification used in our baseline results. It can be seen that when the test set is predicted according to the class with more samples in the training set, the result is 50.0%, which is a completely random result for a binary classification task. In addition, the f1-score is only 0.33. It is because all the samples in the test set are predicted to be of a certain category, and the proportions of the two categories in the test set are exactly the same. Compared with the public datasets, our research obtained a better result based on the use of a balanced dataset, which also shows that our dataset has better quality.

### Results of using deep learning methods

**Figure 5:**
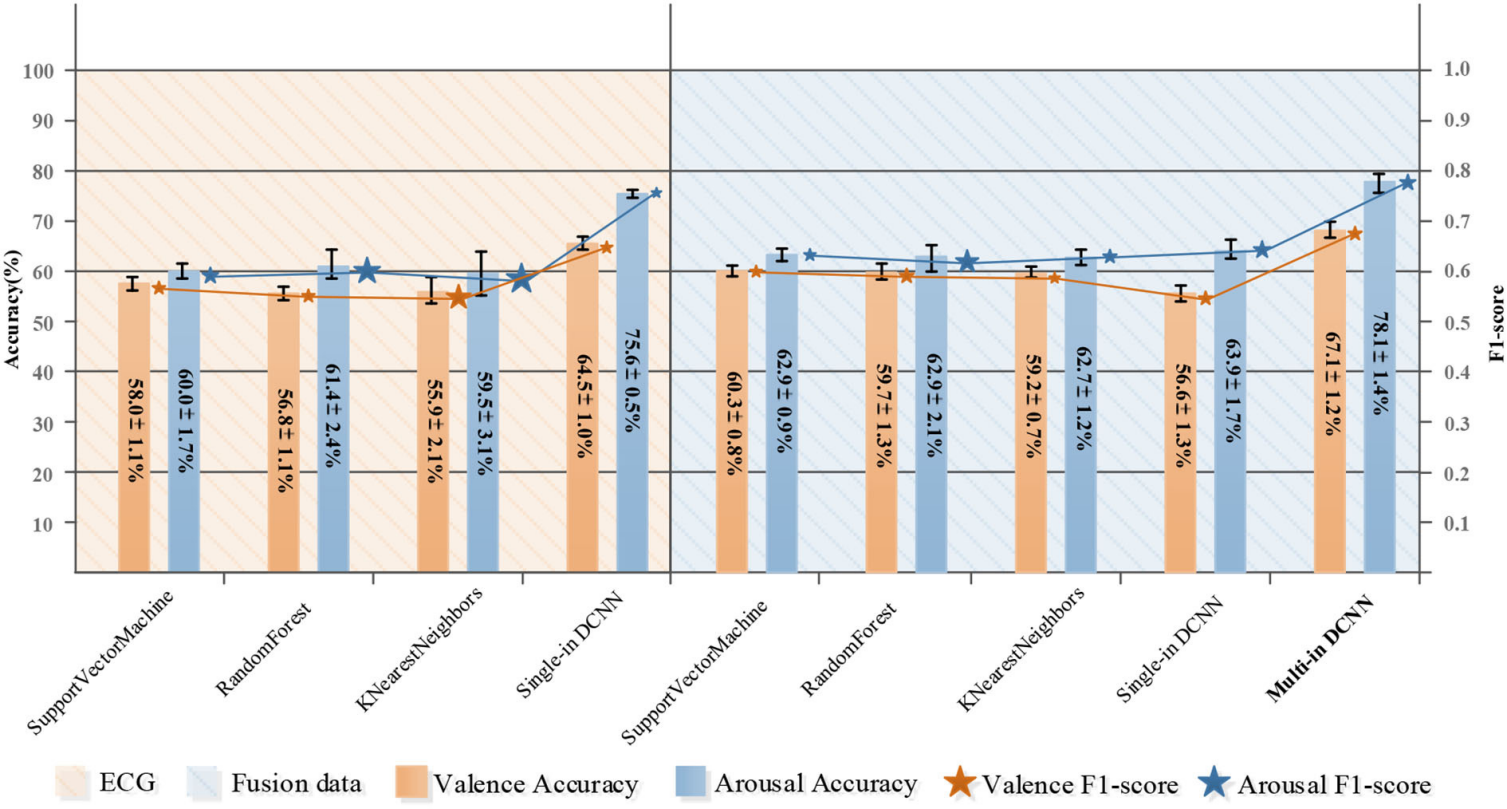
Comparison of emotion classification based on ECG and ECG&EDA&RSP fusion data.

This section discusses the results of our proposed methods. The convolutional neural network is a network structure widely used for image processing. It has been well-applied in computer vision and related fields, but it is still in the exploration stage in the field of emotion recognition using physiological signals. Unlike the three-channel picture information, each type of physiological signal is one unique channel signal; they are independent and represent different functions, so if stacked different physiological signals into one input for convolution like the process for a 2D image, we may not be able to get the effective features of each physiological signal, or even get some meaningless features because in the generation process of each feature map, all the input channels should be considered. Therefore, we introduced the concept of multiple inputs and verified our idea on our collected dataset. Table 7 shows the comparison results of using single-input convolution and multi-input convolution. The first three schemes are based on single-input single-modal, the fourth scheme is single-input multi-modal, and the fifth scheme is multi-input multi-modal. The results show that when the network structure is a single-input DCNN, the classification result of fusion data is worse than that of ECG, which proved that if the single input DCNN is used for different input data, the performance of the classifier may be degraded. However, when using an MI-DCNN, the best results were obtained and compared with the former schemes, and the classification results on valence and arousal were significantly improved, which verified the effectiveness of MI-DCNN. Simultaneously, the fifth scheme also confirmed the superiority of using multi-modality for emotion recognition. We compared the classification performance between traditional machine learning methods and our proposed MI-CNN, and the detailed results are shown in Figure 5. Compared with traditional machine learning methods, our proposed MI-CNN showed a significant improvement in both tasks (*t*-test, arousal: *p* = 9.7E-03 < 0.01, valence: 6.5E-03 < 0.01).

**Table 7:** Results of using our proposed methods in our dataset.

	**Arousal**		**Valence**	
	**Accuracy**	**F1-score**	**Accuracy**	**F1-score**
Single-in DCNN ECG	75.6 ± 0.5%	0.751 ± 0.00	64.5 ± 1.0%	0.642 ± 0.01
Single-in DCNN EDA	58.3 ± 0.5%	0.569 ± 0.01	55.6 ± 0.5%	0.544 ± 0.02
Single-in DCNN RSP	66.2 ± 0.8%	0.656 ± 0.01	58.5 ± 0.4%	0.579 ± 0.01
Single-in DCNN ECG&EDA&RSP	63.9 ± 1.7%	0.635 ± 0.02	56.6 ± 1.3%	0.554 ± 0.02
Multi-in DCNN ECG&EDA&RSP	78.3 ± 1.6%	0.780 ± 0.01	67.1 ± 1.2%	0.666 ± 0.01

**Figure 6:**
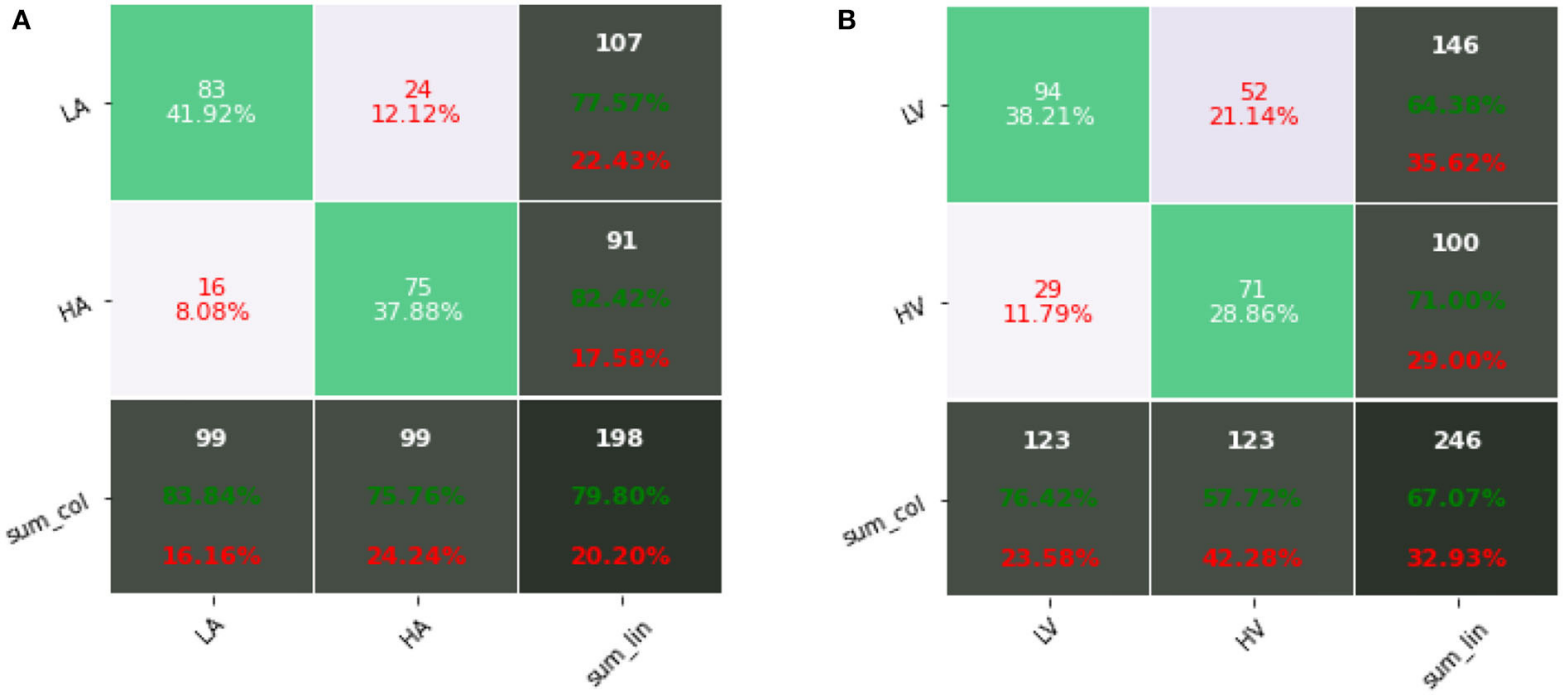
Confusion matrixes using fusion data (ECG&EDA&RSP) of our proposed method: **(A)** arousal; **(B)** valence.

Figure 6 shows confusion matrixes using fusion data of our proposed method, where the row represents the actual label and the column represents the predicted label. Through the confusion matrix, we can observe the specific classification results of our proposed method in each category, including precision and recall. For arousal, the precision of low arousal is 77.57% and recall is 83.84%, and the precision of high arousal is 82.42% and recall is 75.76%. For valence, the precision of low valence is 64.38% and recall is 76.42%, and the precision of high valence is 71.00% and recall is 57.72%. Table 8 shows the comparison of results on two public datasets. The results of our proposed methods were higher than the baseline results (Koelstra et al., 2012; Katsigiannis and Ramzan, 2018). Simultaneously, compared with other end-to-end research (Wang and Shang, 2013), our method also had improvement, which shows a high potential for using the 1D convolutional neural network for automatic emotion recognition tasks.

**Table 8:** Comparison with other research.

**Dataset**	**References**	**Modality**	**End-to-end**	**Arousal**		**Valence**	
				**Accuracy**	**F1-score**	**Accuracy**	**F1-score**
DEAP	(Koelstra et al., 2012)	Peripheral	No	62.7%	0.608	57.0%	0.533
	(Wang and Shang, 2013)	EEG, EOG, EMG	Yes	51.2%	-	60.9%	-
	Our	Peripheral	Yes	67.9%	0.67	68.4%	0.69
DREAMER	(Katsigiannis and Ramzan, 2018)	ECG	No	62.37%	0.5305	62.37%	0.5798
	Our	ECG	Yes	78.6%	0.77	74.7%	0.74

## Conclusion

This work studied the response patterns of physiological signals in different emotions. By designing an emotional induction paradigm, a physiological emotional dataset was constructed, and a complete end-to-end MI-DCNN structure was designed. The results of the MI-DCNN in emotion recognition tasks showed better performance than traditional machine learning methods, and less preprocessing and automatic feature extraction progress showed the MI-DCNN more suitable for automatic emotion recognition. We also studied physiological signal-based emotion recognition from a new perspective, and physiological signals are different from picture information. Considering the application scenario, we propose a multi-input deep convolutional neural network that implements end-to-end automatic emotion recognition based on multi-modal physiological signals. By adding input channels, we solved the problem of interference between channels and achieved the automatic feature extraction step. Simultaneously, by comparing the difference between a single-input CNN and a multi-input CNN, we elaborated on the necessity of using a multi-input CNN when the channel information is quite different. However, some limitations should be noted. First, to obtain a model with stronger generalization ability, the individual difference and temporal difference of biological signals should be considered. Second, we only discussed the benefits of adding input channels to the classification results, ignoring the increase in complexity and parameters they bring to the model. In our future research, we will seek to develop lightweight neural networks that can learn individual and temporal differences from biological signals for automatic emotion recognition.

## Data availability statement

The datasets presented in this article are not readily available because the experiment data is not available online for further research, but available on reasonable request according to the policy of Tianjin University, Capital Medical University, and Tianjin University of Technology. Requests to access the datasets should be directed to CC, cccovb@hotmail.com.

## Ethics statement

The studies involving human participants were reviewed and approved by Beijing Anding Hospital, Capital Medical University. The patients/participants provided their written informed consent to participate in this study. Written informed consent was obtained from the individual(s) for the publication of any potentially identifiable images or data included in this article.

## Author contributions

BZ and CC designed coordinated this research and helped perform the analysis through constructive discussions. PC carried out the experiments and the data analysis and wrote the manuscript. AB, XL, and XZ conceived the study and participated in the research coordination. WY, ZH, and JL contributed significantly to the analysis and manuscript preparation. All authors contributed to the article and approved the submitted version.

## Funding

This research was supported in part by the National Natural Science Foundation of China (61806146, 82101448, 62006014, and U19B2032), the Beijing Municipal Hospital Research and Development Project (PX2021068), the Advanced Innovation Center for Human Brain Protection Project (3500-12020137), the Guangdong Basic and Applied Basic Research Foundation (2021A1515110249), the Scientific and Technological Innovation 2030 (Grant 2021ZD0204300 and 2021Z D0204303), Tianjin Enterprise Science and Technology Commissioner Project under (Grant No. 20YDTPJC00790), and Beijing Key Laboratory of Mental Disorders (Code: 2020JSJB04).

## Conflict of interest

The authors declare that the research was conducted in the absence of any commercial or financial relationships that could be construed as a potential conflict of interest.

## Publisher's note

All claims expressed in this article are solely those of the authors and do not necessarily represent those of their affiliated organizations, or those of the publisher, the editors and the reviewers. Any product that may be evaluated in this article, or claim that may be made by its manufacturer, is not guaranteed or endorsed by the publisher.

## References

[B1] Abadi M. Barham P. Chen J. Chen Z. Davis A. Dean J.. (2016). “TensorFlow: a system for large-scale machine learning,” in Proceedings of the 12th USENIX Symposium on Operating Systems Design and Implementation (OSDI '16) (Savannah, GA), 265–283.

[B2] Abadi M. K. Subramanian R. Kia S. M. Avesani P. Patras I. Sebe N. (2015). DECAF: MEG-based multimodal database for decoding affective physiological responses. IEEE Trans. Affect. Comput. 6, 209–222. 10.1109/TAFFC.2015.2392932

[B3] Abbaschian B. J. Sierra-Sosa D. Elmaghraby A. (2021). Deep learning techniques for speech emotion recognition, from databases to models. Sensors 21, 1249. 10.3390/s21041249 33578714 PMC7916477

[B4] Acharya U. R. Oh S. L. Hagiwara Y. Tan J. H. Adeli H. (2018). Deep convolutional neural network for the automated detection and diagnosis of seizure using EEG signals. Comput. Biol. Med. 100, 270–278. 10.1016/j.compbiomed.2017.09.017 28974302

[B5] Agrafioti F. Hatzinakos D. Anderson A. K. (2012). ECG pattern analysis for emotion detection. IEEE Trans. Affect. Comput. 3, 102–115. 10.1109/T-AFFC.2011.28

[B6] Akhand M. A. H. Roy S. Siddique N. Kamal M. A. S. Shimamura T. (2021). Facial emotion recognition using transfer learning in the deep CNN. Electronics 10, 1036. 10.3390/electronics10091036

[B7] Cacioppo J. T. Tassinary L. G. Berntson G. (2007). Handbook of Psychophysiology. Cambridge: Cambridge University Press.

[B8] Chourasia M. Haral S. Bhatkar S. Kulkarni S. (2021). “Emotion recognition from speech signal using deep learning,” in Intelligent Data Communication Technologies and Internet of Things Lecture Notes on Data Engineering and Communications Technologies, eds J. Hemanth, R. Bestak, and J. I.-Z. Chen (Singapore: Springer), 471–481. 10.1007/978-981-15-9509-7_39

[B9] Cosoli G. Poli A. Scalise L. Spinsante S. (2021). Measurement of multimodal physiological signals for stimulation detection by wearable devices. Measurement 184, 109966. 10.1016/j.measurement.2021.109966

[B10] Gabert-Quillen C. A. Bartolini E. E. Abravanel B. T. Sanislow C. A. (2015). Ratings for emotion film clips. Behav. Res. Methods 47, 773–787. 10.3758/s13428-014-0500-0 24984981 PMC6445277

[B11] Greco A. Valenza G. Lanata A. Scilingo E. P. Citi L. (2016). cvxEDA: a convex optimization approach to electrodermal activity processing. IEEE Trans. Biomed. Eng. 63, 797–804. 10.1109/TBME.2015.2474131 26336110

[B12] Gross J. J. Levenson R. W. (1997). Hiding feelings: the acute effects of inhibiting negative and positive emotion. J. Abnormal Psychol. 106, 95–103. 10.1037/0021-843X.106.1.95 9103721

[B13] Hamilton P. (2002). Open source ECG analysis. Comput. Cardiol. 2002, 101–104. 10.1109/CIC.2002.1166717

[B14] He Z. Li Z. Yang F. Wang L. Li J. Zhou C.. (2020). Advances in multimodal emotion recognition based on brain–computer interfaces. Brain Sci. 10, 687. 10.3390/brainsci,10100687 PMC7600724 33003397

[B15] He Z. Zhong Y. Pan J. (2022). An adversarial discriminative temporal convolutional network for EEG-based cross-domain emotion recognition. Comput. Biol. Med. 141, 105048. 10.1016/j.compbiomed.2021.105048 34838262

[B16] Hjorth B. (1970). EEG analysis based on time domain properties. Electroencephalogr. Clin. Neurophysiol. 29, 306–310. 10.1016/0013-4694(70)90143-4 4195653

[B17] Ioffe S. Szegedy C. (2015). “Batch normalization: accelerating deep network training by reducing internal covariate shift,” in Proceedings of the 32nd International Conference on Machine Learning (PMLR) (Lille), 448–456.

[B18] James W. (1922). The Emotions. Baltimore, MD: Williams & Wilkins Co.

[B19] Katsigiannis S. Ramzan N. (2018). DREAMER: a database for emotion recognition through EEG and ECG signals from wireless low-cost off-the-shelf devices. IEEE J. Biomed. Health Informat. 22, 98–107. 10.1109/JBHI.2017.2688239 28368836

[B20] Kingma D. P. Ba L. J. (2015). “Adam: a method for stochastic optimization,” in International Conference on Learning Representations (San Diego, CA).

[B21] Koelstra S. Muhl C. Soleymani M. Lee J.-S. Yazdani A. Ebrahimi T.. (2012). DEAP: a database for emotion analysis; using physiological signals. IEEE Trans. Affect. Comput. 3, 18–31. 10.1109/T-AFFC.2011.15

[B22] Kolodyazhniy V. Kreibig S. D. Gross J. J. Roth W. T. Wilhelm F. H. (2011). An affective computing approach to physiological emotion specificity: toward subject-independent and stimulus-independent classification of film-induced emotions. Psychophysiology 7, 908–922. 10.1111/j.1469-8986.2010.01170.x 21261632

[B23] Kroenke K. Spitzer R. L. (2002). The PHQ-9: a new depression diagnostic and severity measure. Psychiatr. Ann. 32, 509–515. 10.3928/0048-5713-20020901-06

[B24] Lang P. J. Greenwald M. K. Bradley M. M. Hamm A. O. (1993). Looking at pictures: affective, facial, visceral, and behavioral reactions. Psychophysiology 30, 261–273. 10.1111/j.1469-8986.1993.tb03352.x 8497555

[B25] Liu W. Qiu J.-L. Zheng W.-L. Lu B.-L. (2022). Comparing recognition performance and robustness of multimodal deep learning models for multimodal emotion recognition. IEEE Trans. Cogn. Dev. Syst. 14, 715–729. 10.1109/TCDS.2021.3071170

[B26] Makowski D. Pham T. Lau Z. J. Brammer J. C. Lespinasse F. Pham H.. (2021). NeuroKit2: a Python toolbox for neurophysiological signal processing. Behav. Res. 53, 1689–1696. 10.3758/s13428-020-01516-y 33528817

[B27] Miranda-Correa J. A. Abadi M. K. Sebe N. Patras I. (2021). AMIGOS: a dataset for affect, personality and mood research on individuals and groups. IEEE Trans. Affect. Comput. 12, 479–493. 10.1109/TAFFC.2018.2884461

[B28] Morris J. D. (1995). Observations: SAM: the self-assessment manikin: an efficient cross-cultural measurement of emotional response. J. Advert. Res. 35, 63–68.

[B29] Mustaqeem A. Kwon S. (2021). MLT-DNet: speech emotion recognition using 1D dilated CNN based on multi-learning trick approach. Expert Syst. Appl. 167, 114177. 10.1016/j.eswa.2020.114177

[B30] Pedregosa F. Varoquaux G. Gramfort A. Michel V. Thirion B. Grisel O.. (2018). Scikit-learn: Machine Learning in Python. arXiv [Preprint]. arXiv: 1201.0490. 10.48550/arXiv.1201.0490

[B31] Santamaria-Granados L. Munoz-Organero M. Ramirez-González G. Abdulhay E. Arunkumar N. (2019). Using deep convolutional neural network for emotion detection on a physiological signals dataset (AMIGOS). IEEE Access 7, 57–67. 10.1109/ACCESS.2018.288321335303579

[B32] Siddharth A. Jung T.-P. Sejnowski T. J. (2022). Utilizing deep learning towards multi-modal bio-sensing and vision-based affective computing. IEEE Trans. Affect. Comput. 13, 96–107. 10.1109/TAFFC.2019.2916015

[B33] Soleymani M. Lichtenauer J. Pun T. Pantic M. (2012). A multimodal database for affect recognition and implicit tagging. IEEE Trans. Affect. Comput. 3, 42–55. 10.1109/T-AFFC.2011.2533679348

[B34] Song T. Zheng W. Lu C. Zong Y. Zhang X. Cui Z. (2019). MPED: a multi-modal physiological emotion database for discrete emotion recognition. IEEE Access 7, 12177–12191. 10.1109/ACCESS.2019.2891579

[B35] Subramanian R. Wache J. Abadi M. K. Vieriu R. L. Winkler S. Sebe N. (2018). ASCERTAIN: emotion and personality recognition using commercial sensors. IEEE Trans. Affect. Comput. 9, 147–160. 10.1109/TAFFC.2016.2625250

[B36] Tang H. Liu W. Zheng W.-L. Lu B.-L. (2017). “Multimodal emotion recognition using deep neural networks,” in Neural Information Processing Lecture Notes in Computer Science, eds D. Liu, S. Xie, Y. Li, D. Zhao, and E.-S. M. El-Alfy (Cham: Springer International Publishing), 811–819. 10.1007/978-3-319-70093-9_86

[B37] Tripathi S. Acharya S. Sharma R. D. Mittal S. Bhattacharya S. (2017). “Using deep and convolutional neural networks for accurate emotion classification on DEAP dataset,” in Proceedings of the Thirty-First AAAI Conference on Artificial Intelligence AAAI'17 (San Francisco, California, USA: AAAI Press), 4746–4752. 10.1609/aaai.v31i2.19105

[B38] Verma G. K. Tiwary U. S. (2014). Multimodal fusion framework: a multiresolution approach for emotion classification and recognition from physiological signals. NeuroImage 102, 162–172. 10.1016/j.neuroimage.2013.11.007 24269801

[B39] Wagner J. Kim J. Andre E. (2005). “From physiological signals to emotions: implementing and comparing selected methods for feature extraction and classification,” in 2005 IEEE International Conference on Multimedia and Expo (Amsteram), 940–943. 10.1109/ICME.2005.1521579

[B40] Wang D. Shang Y. (2013). Modeling physiological data with deep belief networks. Int. J. Inf. Educ. Technol. 3, 505–511. 10.7763/IJIET.2013.V3.326 25165501 PMC4142685

[B41] Yildirim Ö. Pławiak P. Tan R.-S. Acharya U. R. (2018). Arrhythmia detection using deep convolutional neural network with long duration ECG signals. Comput. Biol. Med. 102, 411–420. 10.1016/j.compbiomed.2018.09.009 30245122

[B42] Zhang Q. Chen X. Zhan Q. Yang T. Xia S. (2017). Respiration-based emotion recognition with deep learning. Comput. Indus. 92–93, 84–90. 10.1016/j.compind.2017.04.005

[B43] Zhang X. Liu J. Shen J. Li S. Hou K. Hu B.. (2021). Emotion recognition from multimodal physiological signals using a regularized deep fusion of kernel machine. IEEE Trans. Cybernet. 51, 4386–4399. 10.1109/TCYB.2020.2987575 32413939

[B44] Zhang Y. Hossain M. Z. Rahman S. (2021). “DeepVANet: a deep end-to-end network for multi-modal emotion recognition,” in Human-Computer Interaction – INTERACT 2021 Lecture Notes in Computer Science, eds C. Ardito, R. Lanzilotti, A. Malizia, H. Petrie, A. Piccinno, G. Desolda, et al. (Cham: Springer International Publishing), 227–237. 10.1007/978-3-030-85613-7_16

